# Hemoincompatibility in Hemodialysis-Related Therapies and Their Health Economic Perspectives

**DOI:** 10.3390/jcm13206165

**Published:** 2024-10-16

**Authors:** Carsten Hornig, Sudhir K. Bowry, Fatih Kircelli, Dana Kendzia, Christian Apel, Bernard Canaud

**Affiliations:** 1 Fresenius Medical Care Deutschland GmbH, Global Market Access and Health Economics, Else-Kröner-Straße 1, 61352 Bad Homburg, Germany; carsten.hornig@freseniusmedicalcare.com (C.H.); dana.kendzia@freseniusmedicalcare.com (D.K.); christian.apel@freseniusmedicalcare.com (C.A.); 2 Dialysis-at-Crossroads (D@X) Advisory, Wilhelmstraße 9, 61231 Bad Nauheim, Germany; sudhir.bowry@outlook.com; 3 Fresenius Medical Care Deutschland GmbH, Global Medical Office, Else-Kröner-Straße 1, 61352 Bad Homburg, Germany; fatih.kircelli@freseniusmedicalcare.com; 4 School of Medicine, Montpellier University, 34090 Montpellier, France; 5 MTX Consulting, 34090 Montpellier, France

**Keywords:** end stage kidney disease, kidney replacement therapy, hemodialysis, biocompatibility, morbidity, mortality, dialysis-related disease, healthcare economic perspective

## Abstract

Hemobiologic reactions associated with the hemoincompatibility of extracorporeal circuit material are an undesirable and inevitable consequence of all blood-contacting medical devices, typically considered only from a clinical perspective. In hemodialysis (HD), the blood of patients undergoes repetitive (at least thrice weekly for 4 h and lifelong) exposure to different polymeric materials that activate plasmatic pathways and blood cells. There is a general agreement that hemoincompatibility reactions, although unavoidable during extracorporeal therapies, are unphysiological contributors to non-hemodynamic dialysis-induced systemic stress and need to be curtailed. Strategies to lessen the periodic and direct effects of blood interacting with artificial surfaces to stimulate numerous biological pathways have focused mainly on the development of ‘more passive’ materials to decrease intradialytic morbidity. The indirect implications of this phenomenon, such as its impact on the overall delivery of care, have not been considered in detail. In this article, we explore, for the first time, the potential clinical and economic consequences of hemoincompatibility from a value-based healthcare (VBHC) perspective. As the fundamental tenet of VBHC is achieving the best clinical outcomes at the lowest cost, we examine the equation from the individual perspectives of the three key stakeholders of the dialysis care delivery processes: the patient, the provider, and the payer. For the patient, sub-optimal therapy caused by hemoincompatibility results in poor quality of life and various dialysis-associated conditions involving cost-impacting adjustments to lifestyles. For the provider, the decrease in income is attributed to factors such as an increase in workload and use of resources, dissatisfaction of the patient from the services provided, loss of reimbursement and direct revenue, or an increase in doctor–nurse turnover due to the complexity of managing care (nephrology encounters a chronic workforce shortage). The payer and healthcare system incur additional costs, e.g., increased hospitalization rates, including intensive care unit admissions, and increased medications and diagnostics to counteract adverse events and complications. Thus, hemoincompatibility reactions may be relevant from a socioeconomic perspective and may need to be addressed beyond just its clinical relevance to streamline the delivery of HD in terms of payability, future sustainability, and societal repercussions. Strategies to mitigate the economic impact and address the cost-effectiveness of the hemoincompatibility of extracorporeal kidney replacement therapy are proposed to conclude this comprehensive approach.

## 1. Introduction

Hemodialysis is a lifesaving therapy that is recognized as the primary line of treatment in the management of end-stage kidney disease (ESKD) patients, and is daily used by over 3 million patients worldwide [1,2,3]. However, as an active and intrusive therapy, hemodialysis carries inherent hazards, including adverse events, side effects, and complications, that contribute to the overall disease and therapeutic burden faced by ESKD patients [4,5,6]. Among these hazards, hemoincompatibility, a marker of adverse reactions between the patient’s and dialysis components, plays a significant role in altering patient outcomes and increasing healthcare costs depending on the dialyzer of choice [7,8,9,10,11,12]. Despite this established link, the health economic consequences of hemoincompatibility in hemodialysis or hemodialysis-related therapies (i.e., hemodiafiltration) remain unexplored.

In this narrative essay, we aim to briefly review the mechanisms of hemoincompati-bility from a pathophysiological perspective, analyze the clinical outcomes and health-related complications associated with hemoincompatibility, assess the economic burden of hemocompatibility on the healthcare system, delineate strategies to mitigate the occurrence of hemoincompatibility, and provide a brief and practical conclusion.

## 2. Understanding Hemoincompatibility and Their Mechanisms

**Figure 1:**
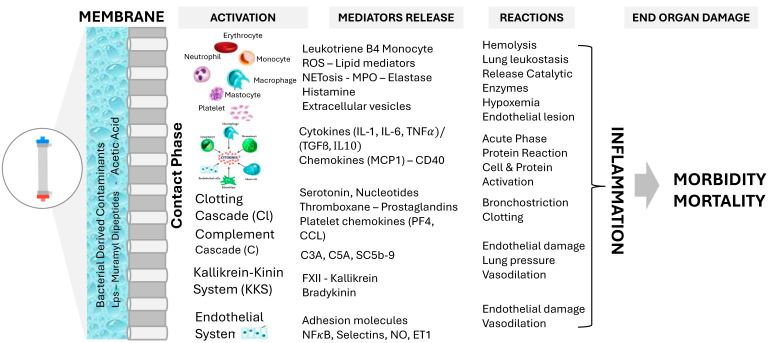
Hemoincompatibility reactions induced by membrane contact and dialysis fluid contaminants result in the activation of various protein cascades and cells, leading to mediator release, reactions, and organ damage; ROS, reactive oxygen species; NETosis, Neutrophil Extracellular Trap Formation; MPO, myeloperoxidase; C3a, C5a, SC5b-9, Complement fractions; NFkB, Nuclear Factor-kappa B; NO, Nitric Oxide; ET1, Endothelin 1.

Hemoincompatibility, or more broadly bioincompatibility, refers to a range of adverse biological reactions that occur when blood interacts with the extracorporeal circuit of a hemodialysis treatment, reflecting the bioreactor properties of the dialyzer (Figure 1) [10,13,14,15]. These reactions, initiated by contact with extracorporeal foreign material (such as needles, tubing lines, dialyzers, and bubble traps), include the activation of various protein cascade systems (such as clotting, complement, and kallikrein–kinin) [16,17]. Additionally, there is an activation of circulating cells (such as leukocytes, platelets, lymphocytes, and erythrocytes) or immobilized cells (such as endothelial cells), which may release various soluble mediators (such as enzymes, cytokines, and reactive oxygen species). These mediators contribute to the enhancement, propagation, and systemic extension of these biological reactions through various amplification loops, ultimately inducing end organ damage and contributing to clinical or subclinical reactions [10,18,19].

More importantly, it has been evidenced that microbial contaminants of dialysate (such as endotoxins) or chemical components (such as acetate) contribute to enhance these hemobiologic reactions [20]. The endotoxin retention capacities of the dialyzers used will help limit this interaction, thereby reducing the burden. As this phenomenon follows repeated cycles of intermittent hemodialysis, typically three times weekly or more frequently, hemoincompatibility reactions tend to be sustained over time. This contributes to maintaining a subclinical inflammatory state, recognized as a significant contributor to non-hemodynamically induced systemic stress in dialysis patients, leading to various end organ damages [17,21,22]. This is briefly schematized in Figure 1. 

More specifically, the next section will briefly detail these hemobiologic reactions schematically, along with their time course of occurrence: 

### 2.1. Contact Phase and Protein Deposition

**Figure 2:**
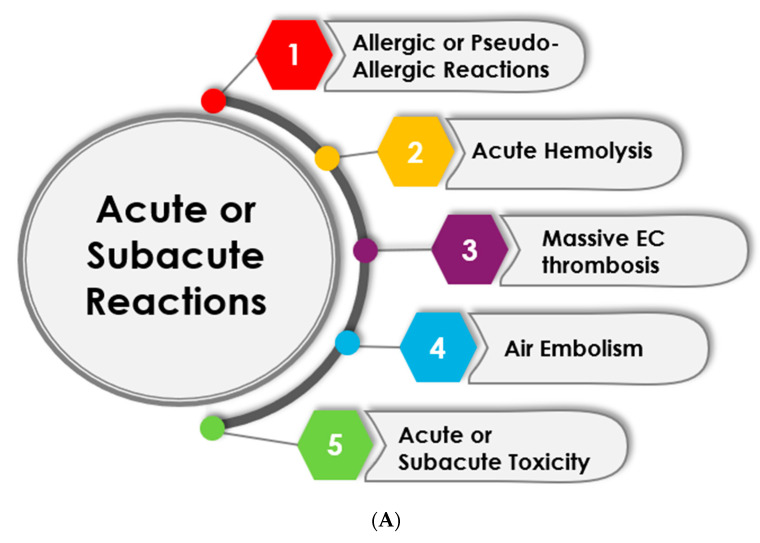

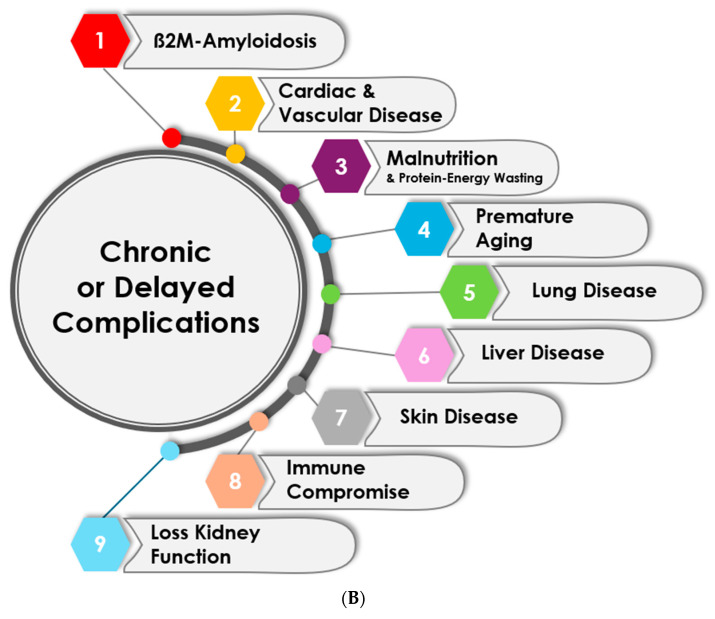
(**A**) Clinical outcomes and health implications of acute or subacute hemoincompatibility reactions induced by hemodialysis. (**B**) Clinical outcomes and health implications of chronic or delayed complications of hemoincompatibility reactions induced by hemodialysis.

In the contact phase activation of blood with artificial surfaces, protein adsorption is the initial event, occurring almost immediately and covering the surface within seconds (Figure 2) [23]. This adsorbed protein layer influences subsequent interactions and alterations of blood components. The material’s surface chemistry and properties, such as hydrophobicity, charge, and nanostructures, affect which proteins adsorb and how they behave [16]. These properties, combined with thermodynamic forces and kinetics, determine the conformation and stability of protein adsorption [19,24]. In hemodialysis (HD), early unmodified cellulosic membranes benefit from protein adsorption, which passivates the surface, reducing adverse reactions. However, with the advent of more biocompatible or surface-modified synthetic membranes, such passivation is no longer necessary [19]. Some dialysis membranes adsorb more proteins, which may be beneficial for uremic toxin removal (i.e., ß2M, Protein Bound Uremic Toxins), but this does not necessarily enhance hemocompatibility. High levels of protein adsorption can hinder solute removal during HD or more importantly in convective-based therapies (i.e., HDF). Recent findings suggest that stable solute clearance is achievable throughout HD/HDF therapy, contrary to the usual performance decline due to protein build-up on the membrane.

### 2.2. Activation of the Clotting System, Including Platelet Activation

The activation of the clotting cascade in extracorporeal circuits is the first visible sign of system hemoincompatibility [25,26]. The main pathways initiating blood coagulation are the tissue factor and contact (factor XII) activation pathways, which activate factor X to Xa, leading to thrombin formation and ultimately fibrin production. This process then comes with the conversion of fibrinogen to fibrin by thrombin, representing the final step of these series of reactions. In blood-contacting extracorporeal circuits, contact activation is critical, as different materials trigger biological pathways differently. Surface parameters such as chemistry, energy, and wettability influence the initial contact activation of coagulation. Research has focused on factor XII-dependent initiation of coagulation by various dialysis membranes to develop those with reduced coagulation potential. Heparin (unfractionated or fractionated) is currently the most widely used agent to inhibit the coagulation cascade but does not fully prevent the generation of thrombin–antithrombin III complexes, prothrombin F1+2, and D-dimers. A recent study has focused on the terminal phase of coagulation cascades and highlighted that the imbalance of factor XIII (Fibrin Stabilizing Factor) and alpha (2)-plasmin inhibitor (a2PI) is a potential additional cause of increased thrombosis risk in HD [27]. These are markers of thrombin activity on fibrinogen produced in extracorporeal circuits, even with heparin anticoagulation. This is why various alternative anticoagulation modalities are currently being explored, such as direct oral anticoagulants, heparin-coated circuits, and citrate/calcium infusion.

Platelet activation and its association with blood coagulation are crucial in extracorporeal circuits [28,29,30]. The primary step involves the adhesion of platelets to surfaces, either directly or via adsorbed proteins like fibrinogen and the von Willebrand factor. Certain material properties, such as negative charge, enhance this adhesion, while adsorbed albumin discourages it. Once adhered, platelets change shape, spread, and release granule contents, including ß2-thromboglobulin and platelet factor 4 [31]. Surface receptors like GPIIb/IIIa facilitate further platelet interactions and aggregation. These processes expose phospholipids that aid in the binding of coagulation proteins, promoting clot formation. In hemodialysis, the sequence of platelet adhesion, activation, aggregation, and clot formation are significant markers of hemoincompatibility [30].

### 2.3. Activation of the Complement System

The activation of the complement system resulting from membrane–blood interactions is mediated mainly through the alternate pathway (C3b, Properdin) and the lectin pathway (ficolin) [32,33]. This leads to the early production of C3a, and later C5a and sC5b9, which act as anaphylatoxins [34]. Complement activation and its consequences have significantly impacted the development of dialysis membranes [34,35,36]. It is widely acknowledged as undesirable because it is potentially associated with severe adverse events (e.g., first-use syndrome, lung dysfunction, hypoxemia, intradialytic morbidity) and subclinical long-term events (e.g., vascular disease), contributing to increased morbidity in dialysis patients and higher costs of dialysis [37]. Historically, unmodified cellulosic-based membranes, once prevalent in hemodialysis, were phased out due to the high activation of the complement system’s alternative pathway. Research led to the development of more biocompatible membranes, starting with modified cellulosic (reducing or masking hydroxyl radicals) and then synthetic polymer-based membranes [38]. Despite improvements, synthetic membranes still exhibit low levels of complement activation [39]. This residual complement activation has raised new concerns, as it has been associated in recent studies with short- and/or mid-term severe cardiac events [40]. As such, new efforts have been focused on membrane development to mitigate or abolish this complement activation [41].

### 2.4. Activation of the Kallikrein–Kinin System

During hemodialysis, contact with the dialyzer membrane surface, depending on their physical (e.g., electric charges) or chemical characteristics, can activate the kallikrein–kinin system through both the contact phase and complement activation [42]. This activation leads to the production of bradykinin, a molecule with complex and paradoxical effects on the vascular and bronchoalveolar systems [43]. Bradykinin can cause vasodilation and severe hypotension, but in some cases, it can induce paradoxical hypertension. It plays a role in both inflammatory responses and blood pressure regulation by acting on endothelial cells. Furthermore, bradykinin has been implicated in severe allergic reactions experienced by hemodialysis patients, especially those taking ACE inhibitors [44,45,46].

### 2.5. Activation of Blood Cells, NET Formation, Release of Mediators

The leukocyte complement activation axis plays a crucial role in hemoincompatibility reactions during hemodialysis (HD). Complement activation during HD mirrors leukopenia, with the intensity influenced by the nature and properties of the dialysis membranes (cellulosic or synthetic) [32]. Leukocytes bind to C3 fragments on the membrane surface (mostly hydroxyl radicals), leading to leukopenia and the further recruitment, activation, and degranulation of neutrophils, which trigger oxidative burst [47,48]. Additionally, circulating monocytes may be activated either by contact with the membrane or by endotoxins present in the dialysate, initiating the synthesis and release of proinflammatory cytokines (IL-1, IL-6, TNFα), which actively contribute to the acute phase reaction [49,50].

Recently, it has been proposed that the formation of Neutrophil Extracellular Traps (NETs), known as NETosis, may serve as an integrated marker of hemodialysis bioincompatibility reactions [51,52]. NETs result from neutrophil degranulation induced by reactive oxygen overproduction via NADPH oxidase. They consist of modified chromatin, decorated with serine proteases, elastase, bactericidal proteins, and myeloperoxidase (MPO), which produces hypochlorite anion. Byproducts of NETs, such as elastase, MPO, and cellfree DNA, have been reported to increase in hemodialysis patients, particularly during dialysis sessions [52]. Furthermore, as NETs and MPO can be taken up by the endothelium, NETs could be considered a vascular memory of the intermittent bioincompatibility phenomenon [51]. Interestingly, NETs have recently been identified as a major harmful component in a wide range of pathologies, including vascular diseases associated with inflammatory processes. This indicates that NETs could be a significant marker to focus on in HD patients.

### 2.6. Activation of Endothelial Cells and Release of Extracellular Microvesicles

Endothelial injury is part of the complex bioincompatibility reactions induced by the hemodialysis (HD) system [53]. Endothelial cells respond to this stress by shedding endothelial-derived extracellular microvesicles (EMVs) or microparticles with proinflammatory and pro-coagulant activities, as well as a propensity for inducing vascular lesion and calcification [54,55,56]. Studies have shown that the number and size of circulating EMVs (marked by CD144+) are significantly higher in HD patients compared to healthy individuals or non-dialysis CKD patients [54]. Furthermore, EMVs increase and modify their size during HD sessions, varying according to the dialysis modality. In this context, hemodiafiltration (HDF) and convective-based therapies are beneficial in mitigating the increase in and modulating the size of EMVs [53,57,58,59].

### 2.7. Activation of Acute Phase Protein Synthesis and Inflammation

The activation of various protein cascades (coagulation, complement, kinin–kallikrein) and circulating cells (platelets, leukocytes) induced by contact with the extracorporeal circuit triggers the release of proinflammatory mediators (cytokines, granulocyte enzymes) and reactive oxygen species from leukocytes, ultimately leading to oxidative stress and inflammation [48,49]. These reactions amplify and extend the process, inducing the production of acute phase reactants by the liver in a broader reaction called inflammasome production [60]. Microbial impurities (e.g., endotoxin, LPS, muramyl dipeptides) in the dialysis fluid can enhance and catalyze these hemobiological reactions [61]. The repeated cycles of hemoincompatible phenomena, triggering and maintaining low grade inflammation, contribute to end organ damage and increased mortality [62,63].

All these biological reactions occur in the hemodialyzer, and extracorporeal circuits interact with each other, creating a vicious cycle with amplification loops that propagate systemically in circulation and maintain them beyond the duration of dialysis. The crosstalk between major players is well documented for the interplay between complement and coagulation, proinflammatory cytokines and oxidative stress, as well as between coagulation activation and proinflammatory cytokines, and between granulocyte activation with enzyme release and proinflammatory cytokine release or endothelial damage [62,64,65,66,67]. Additionally, the intensity of these reactions can vary between patients due to individual sensitivity, as well as over time, depending on procedural conditions, patient health status, and other unexpected factors. Along with uremic disorders, these hemobiologic reactions contribute to end organ damage, particularly affecting the cardiovascular system and leading to cardiovascular disease [68].

## 3. Clinical Outcomes and Health Implications

Hemoincompatibility reactions during hemodialysis encompass, but are not limited to, issues with the extracorporeal blood circuit, water and dialysate contaminants, and IV drug administration. These reactions have diverse clinical consequences, impacting various organs with varying severity at different points within the patient’s treatment cycle. These consequences can be broadly categorized as either acute/subacute reactions or chronic/delayed complications.

### 3.1. Acute and Subacute Reactions

These reactions occur during or shortly after dialysis and often present as life-threatening complications [69]. This is briefly schematized in Figure 2A. Examples are as follows:

**Allergic or pseudo-allergic reactions**: These reactions may occur almost immediately or early during an HD session. Clinical manifestations are diverse (e.g., malaise, chills, pruritus, hypotension, tachycardia, shortness of breath, wheezing, abdominal pain) and can range in intensity from modest to life-threatening [37]. Several components of the extracorporeal circuit have been implicated (e.g., ethylene oxide, formaldehyde, polymers, or plasticizers) or putatively suspected (e.g., Polysulfone) in triggering these reactions. These reactions may reflect true allergic responses mediated by IgE (e.g., ethylene oxide) [37,70,71] or pseudo-allergic responses mediated by different pathways [72], including complement activation (CARPA) [73,74]. The role of plasticizers embedded in synthetic polymers, such as bisphenol A (BPA) [75], polyvinylpyrrolidone (PVP), or phthalates, has been suggested as a potential cause of sensitization [76]. Additionally, the role of the sterilization process (e.g., gamma radiation) in polymer sensitization has been recently advocated [77].

**Acute hemolysis**: This is due to the rapid destruction of red blood cells within the extracorporeal circuit due to osmotic (electrolytic composition), chemical (toxic component, lead, cupper, chloramine) or mechanical stress [78]. Depending on the intensity of the hemolysis, the symptomatology may vary from simple malaise, back pain, chills, and hypotension, to severe hypotension or shock.

**Massive extracorporeal thrombosis**: Thrombosis of the extracorporeal circuit within the course of dialysis is due to inadequate anticoagulation, the antagonization of heparin (e.g., acidosis), or resulting from heparin-induced thrombopenia [79,80].

**Air embolism**: Air bubbles may be formed in the extracorporeal bloodstream due to various reasons (e.g., partial disconnection within the negative pressure segment of circuit, negative pressure exerted by the blood pump on blood, gas formation within dialysate side) [81,82,83,84,85]. Microbubbles can cause symptoms such as cough, chest pain, shortness of breath, shock, and stroke. Massive or large microbubble air embolism may cause sudden death [83]. A particular severe and lethal outbreak of sudden death through gas embolism has been reported from dialyzer contamination with unemulsified perfluorocarbon (PFC), which is used for testing repair dialyzers [86,87].

**Acute or subacute toxicity**: Contamination of water and dialysate may cause sudden death, usually as outbreaks. These may come from chemical toxicity (e.g., aluminum, chloramine, fluoride, copper, formaldehyde) [88,89,90] but also from microorganisms such as microcystin produced by the blue–green alga Microcystis Aeruginosa [91,92,93]. Patients develop acute neurotoxicity or subacute hepatotoxicity, with symptoms ranging from nausea and vomiting to blindness and convulsions and sudden death.

### 3.2. Chronic and Delayed Complications

These complications develop over months or years of hemodialysis therapy and contribute to dialysis-associated diseases. This is briefly schematized in Figure 2B. Examples are as follows:

**Beta-2 Microglobulin amyloidosis**: Beta-2-Microglobulin amyloidosis (ß2MA) is a disabling condition that can affect long-term hemodialysis patients. Identified in the 1990s, it is characterized by the accumulation, deposition, and transformation of ß2M under local conditions into amyloid fibrils in synovial and osteoarticular tissues in patients with end-stage kidney disease due to high levels of circulating ß2M [94,95]. As a result, it causes destructive osteoarthropathies, such as carpal tunnel syndrome, flexor tenosynovitis, subchondral bone cysts, and erosions, as well as pathological fractures. The most severe complication involving ß2MA deposits is the destruction of paravertebral ligaments and intervertebral discs, which can result in paraplegia [96,97,98,99]. Visceral involvement has also been found in various sites, including the gastrointestinal (GI) tract, heart, and tongue, as well as cardiac involvement, leading to fatal arrhythmias. Several epidemiologic and interventional studies have confirmed that ß2MA was related to ß2M accumulation resulting from the combined use of low flux membranes that do not clear ß2M and bioincompatible cellulosic membranes that stimulate its production via complement activation. This reaction is amplified by contaminated dialysate fluid, resulting in a microinflammatory state. Interestingly, the increased use of high-flux permeable membranes with ultrapure dialysis fluid has almost eliminated ß2MA by more efficiently clearing ß2M and mitigating complement and inflammation reactions [100,101].

**Accelerated vascular disease**: Accelerated atherosclerosis was recognized in hemodialysis patients over 50 years ago [102]. From this initial clinical observation, it has been consistently established that chronic kidney disease is a major cardiovascular risk factor aside from traditional ones, beginning early during kidney disease progression [103]. In this context, hemodialysis has been confirmed as a significant disease modifier and enhancer of atherosclerosis and vascular disease risk. Among the pathogenic factors contributing to vascular disease progression, the hemoincompatibility of the extracorporeal circuit, evidenced by residual complement activation and subclinical chronic inflammation, has been implicated [40,103,104,105,106]. Additionally, the accumulation of uremic toxins (such as middle molecular weight toxins like ß2M and protein-bound uremic toxins like indoxyl sulfate), fluid retention and hypertension, and metabolic abnormalities resulting from the uremic milieu (such as hyperphosphatemia, bone mineral disorders, and lipid disorders) are strong contributive factors to these vascular lesions [107]. Although it remains difficult to disentangle the precise roles of hemoincompatibility and dialysis treatment efficiency in the atherosclerosis process, observational and interventional studies have shown that using high-flux biocompatible membranes, ultrapure dialysate, and intensifying solute removal (such as hemodiafiltration) might have a protective effect on the vascular system of dialysis patients [108,109].

**Cardiovascular disease:** Cardiovascular disease (CVD) is highly prevalent among dialysis patients, with over 80% presenting with at least one form of CVD at the onset of dialysis. The most common cardiovascular conditions in this group include the following: left ventricular hypertrophy (29–75%), congestive heart failure (20–40%), coronary artery disease (22–39%), arrhythmias, including atrial fibrillation (11–27%), sudden cardiac death (15–24%), and valvular heart disease (24%), particularly aortic stenosis [110,111,112,113,114]. The high burden of CVD in patients with end-stage renal disease (ESRD) is driven by both traditional risk factors such as hypertension and diabetes, as well as non-traditional factors specific to kidney disease, including altered bone mineral metabolism, endothelial dysfunction, volume overload, and uremic toxins [111,112]. ESRD patients have a 16- to 19-fold higher mortality rate compared to the general population, with cardiovascular causes responsible for over 50% of deaths [114,115,116]. Hemodialysis itself, through dialysis-induced systemic stress, adds to the cardiovascular strain in this already vulnerable population [11]. Repeated myocardial injury and stress due to fluid shifts, electrolyte imbalances, and exposure to uremic toxins during hemodialysis sessions likely contribute to the high rates of arrhythmias, heart failure, and sudden cardiac death seen in these patients. Early screening for cardiovascular disease is crucial to identify complications sooner and reduce risk. The aggressive management of both traditional and kidney-specific CVD risk factors, along with the optimization of dialysis prescriptions, can help lower cardiovascular risk in this high-risk population. In this context, hemoincompatibility reactions, which are mediated by inflammation, are also recognized contributors to cardiovascular disease, such as non-hemodynamic dialysis-induced systemic stress [21,117].

**Malnutrition and protein energy wasting**: Protein energy malnutrition is recognized as a leading cause of morbidity and mortality in dialysis patients. The protein energy wasting process is observed in 40 to 50% of the dialysis population, depending on the biomarkers used [118,119]. Loss of lean tissue mass and sarcopenia are highly prevalent in this population, leading to frailty. Although several factors have been identified in protein energy wasting, including the retention of uremic toxins, acidosis, fluid overload, and/or losses of amino acids and proteins during dialysis, it has been well established that hemodialysis per se [105,106,120], through hemoincompatibility reactions including complement activation, inflammation, and oxidative stress mechanisms, plays a central role in muscle catabolism [121,122,123] involving mitochondrial dysfunction aggravated by intracellular phosphate depletion [124,125,126]. Observational and interventional studies using high-flux synthetic membranes with a more hemocompatible profile, ultrapure dialysis fluid, and increased uremic solute clearance (such as hemodiafiltration) have been able to mitigate muscle degradation as well as preserve nutritional status including lean tissue mass, and biomarkers such as albumin and transthyretin [127,128,129,130].

**Premature aging phenomenon**: Premature aging in dialysis patients is a well-recognized and documented phenomenon [131]. Aging is a multifactorial process influenced by uremic abnormalities, including the accumulation of uremic toxins and immune dysfunction, which progress with kidney disease [131,132,133]. However, it is further accelerated by kidney replacement therapy, indicating that hemodialysis plays a role in this phenomenon. Recognizing that residual complement activation, oxidative stress, inflammation, and the formation of advanced glycation end products (AGEs) associated with hemoin-compatible dialysis are major contributors to cellular senescence, it is easy to postulate that aging is precipitated by these hemobiologic reactions [134]. Even though no specific study has objectively explored biological reactions associated with aging in hemodialysis patients, one may speculate that using high-flux biocompatible dialyzers, ultrapure dialysate, and enhanced clearances of middle and large molecular weight uremic compounds will be beneficial in mitigating this senescent phenomenon [135].

**Lung disease:** Lung disease or respiratory disorders as well as pulmonary hypertension are very prevalent complications in hemodialysis patients [136,137,138,139]. The pathogenesis of impaired pulmonary functions is not completely elucidated but it involved, aside fluid overload and congestive heart failure, repetitive hemoincompatibility reactions and microembolism as part of inflammation and fibrosis processes that alter gas exchange and impair respiratory function [136,137]. Recently, it has been clearly shown that chronic hypoxia present in about 10% of HD patients was associated with poor outcomes [140,141]. In addition, tissular alterations resulting from hypoxia are prone to lung disease but also to various tissular injuries [142].

**Liver disease**: Liver disease is a common complication in about one third of HD patients. It mostly caused by hepatitis (B, C) or its sequalae, or hepatotoxic drugs or illness [143]. However, plastic particles can accumulate in the macrophages of the liver and spleen as part of plastic spallation from dialysis tubing (silicone, PVC, polyurethane) [144].

**Skin disease**: Skin problems are quite common in hemodialysis (HD) patients. These typically include dryness and itching (xerosis), hyperpigmentation, pruritus (itching), purpura, nail fragility, and abnormalities as part of uremic syndrome [145,146]. Bullous dermatosis has been reported as part of the syndrome of cutaneous fragility and blistering occurring in HD patients associated with abnormal porphyrin metabolism [147,148]. This can be also likely attributed to hemoincompatibility reactions associated with HD.

**Immune compromise:** Immune disorders are a hallmark of hemodialysis patients. Immune disturbances involve both the innate and adaptive immune systems, explaining their higher susceptibility to infections and deficiencies in immune response to vaccination [149,150]. These immune disorders are linked to uremic retention solutes and are strongly associated with hemoincompatibility reactions, which contribute to a state of subclinical chronic inflammation and an imbalance between pro- and anti-inflammatory mechanisms [151,152].

**Loss of kidney function:** Loss of native kidney function is a common feature in hemodialysis patients after a few months of treatment that affects patients’ outcomes [153]. It reflects dialysis-induced systemic stress in both hemodynamic and non-hemodynamic components. Hemoincompatibility reactions are significant contributors to inflammation and fibrosis kidney lesions. The use of improved hemocompatible and more efficient convective-based therapies tend to reduce the speed of loss of kidney function.

## 4. Economic Burden on Healthcare System

**Figure 3:**
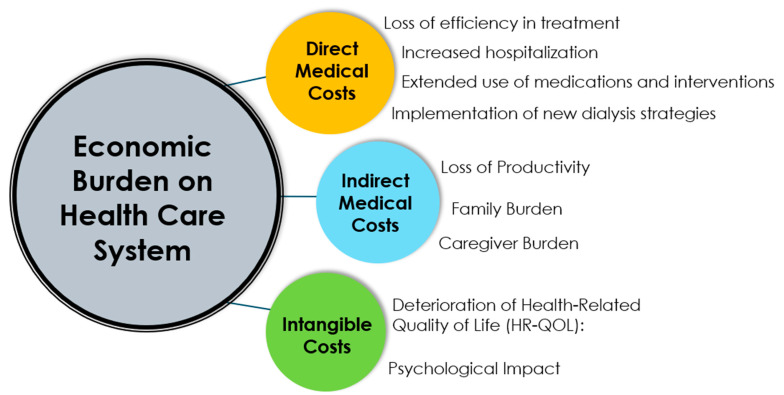
Economic burden of bioincompatibility reactions associated with chronic hemodialysis.

The economic impact of hemoincompatibility can be substantial in the overall cost of kidney replacement therapy, even though it is difficult to disentangle efficiency and hemocompatibility [154,155,156]. This impact includes direct medical costs, indirect costs, and intangible costs. This is briefly schematized in Figure 3.

### 4.1. Direct Medical Costs

Direct medical costs affect the healthcare system in various ways and can be categorized as follows:

**Loss of efficiency in treatment**: Suboptimal blood purification due to hemoincompatibility reactions, either by reducing solute clearances or shortening treatment time to accommodate the patient’s condition, may result in inadequate treatment delivery. This contributes to increased morbidity, higher hospitalization rates, and a negative impact on the patient’s treatment burden [157]. Additionally, inadequate treatment delivery may negatively impact the reimbursement of care providers. In some countries, reimbursement systems for dialysis are based on the attainment of distinct clinical treatment targets for patients. All dialysis facilities must regularly document defined targets, such as the delivered dose of dialysis, dialysis frequency (thrice weekly), treatment time (240 min/session), and hemoglobin levels (10 g/dL). If quality targets are not met, stepwise sanctions may become effective. Patient outcome-related reimbursement schemes are increasingly implemented in most countries, and reported poor outcomes are linked to reduced reimbursement. For example, in the US, within the Quality Incentive Program (QIP), dialysis facilities that do not meet certain standards are subject to a global Medicare payment reduction of up to 2%.

**Increased hospitalization**: Higher hospitalization rates and longer stays, including admissions to the intensive care unit (ICU), are due to complications such as fluid overload, cardiac events, infections, malnutrition, anemia, and various other uremic-related complications [158,159,160,161]. In this context, the increased use of medical imaging or laboratory testing significantly contribute to additional healthcare expenditures [162,163]. When looked from an economic perspective, although there is a general scarcity of cost–utility analysis data in dialysis, three studies examined the additional costs incurred by the procedure-related hospitalization of HD and PD patients: the cost range of increased complication-related hospitalizations was between EUR 1609 and 2772 (range reported is derived from the three publications cited and costs are reported in EUR at a 2021 price level, i.e., including inflation) [164,165,166]. Further to this, costs for HD treatments conducted in hospitals are mostly covered by specific reimbursement schemes that incur higher costs to the healthcare system. Two countries having specific reimbursement schemes are Germany (2021 Reimbursement Code 8-854.3) and the United Kingdom [167,168].

**Extended use of medications and interventions**: The increased use of antibiotics to treat blood stream-related infections, specific medications (antalgic, steroids, cardiac medications) to treat hypersensitivity or intercurrent illnesses, or the use of enteral or parenteral nutrition to treat malnutrition represents significant additional costs for the healthcare system. Extensive imaging exploration (e.g., ultrasound, CT scan, MRI), which is required in this context, also places a significant economic burden on the healthcare system. For example, the consumption of erythropoietic stimulating agents (ESAs) and IV iron use may be reduced by 20 to 30% while keeping hemoglobin levels in the target range when high volume HDF is applied, confirming that the use of more efficient and biocompatible systems has an impact on medication costs [169,170,171].

**Implementation of new dialysis strategies**: New therapeutic approaches, including short-term rescuing procedures like isolated ultrafiltration and intensive hemodialysis with daily treatment programs, have been adopted. Ultimately, transitioning patients to other dialysis modalities as necessary is part of the economic burden for the healthcare system as well as the therapeutic burden for patients [172].

### 4.2. Indirect Medical Costs

Indirect medical costs predominantly impact society and can be categorized as follows:

**Loss of Productivity**: Reduced productivity among active workers or in domestic tasks due to illness is difficult to quantify but is clearly part of the additional cost burden on the healthcare system, industry, and employers.

**Family Burden**: Managing and supporting a HD patient is associated with increased responsibilities and stress for family members [173], which may be accentuated in the case of home HD treatment.

**Caregiver Burden**: Increased demands on caregivers, such as nurses in in-center dialysis facilities, to increase productivity or to more carefully track daily care with digital tools, may lead to physical and emotional strain, potentially resulting in burnout syndrome [174].

In this context, it is of paramount importance to minimize and prevent most hemoincompatible reactions to mitigate this additional risk and burden on HD patients.

### 4.3. Intangible Costs

Intangible costs associated with hemoincompatibility reactions primarily affect patients’ perception of their treatment and add to the overall burden of their kidney disease. These include the following:

**Deterioration of Health-Related Quality of Life** (HR-QOL): A very common pattern in HD patients is a slow decline in various domains of their quality of life over time. This includes physical health, mental well-being, social activity, and the burden of treatment. This decline can even predict patient outcomes [175,176]. Some treatment options, such as the correction of anemia by ESA and iron [177], using home treatment [178,179], or even more recently, high volume HDF [180], have been identified to potentially mitigate this decrease in HD-QOL.

**Psychological Impact**: Hemodialysis treatment is well-recognized to significantly impact mental health, leading to increased depression and psychological distress. This aspect should be carefully monitored and managed because it has a significant impact on both patient-reported outcomes (PROs) and patient-reported experiences (PREs).

Therefore, it is now recommended to more closely monitor patient-reported outcomes (PROs) or patient-reported experiences (PREs) as metrics of treatment adequacy, particularly in the context of hemodialysis and potential hemoincompatibility reactions.

## 5. Strategies to Mitigate Economic Impact and Address Cost-Effectiveness

**Figure 4:**
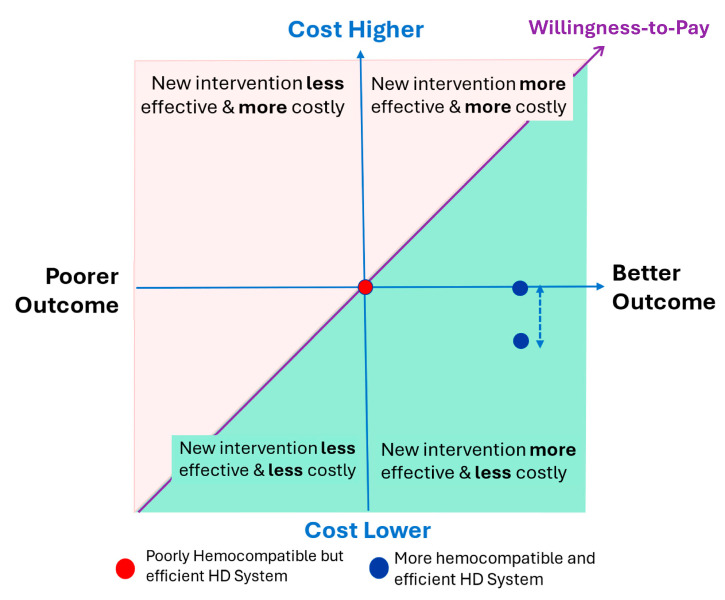
The cost-effectiveness plane for addressing hemodialysis-related hemoincompatibility reactions. Compared to a poorly hemocompatible HD system with high efficiency (red point in the middle), an enhanced hemocompatible HD system with high efficiency such as HDF (blue points) would result in better outcomes at lower or equivalent costs.

By improving the hemocompatibility of the entire extracorporeal treatment component, patients, payers, providers, and suppliers could all benefit, ensuring economic sustainability for the healthcare ecosystem. In this context, the cost-effectiveness plane is an important tool that enables payers to assess the value and cost-effectiveness of a new intervention or device. The example given in the Figure 4 depicts the potential benefits of having a more efficient and hemocompatible extracorporeal treatment system compared to the current standard. Any system with improved hemocompatibility and efficiency, as described below, could then be placed on the right side of the plane. Health technology assessment (HTA) agencies would favor treatment systems that are in the lower right quadrant of the cost-effectiveness plane. Significantly, such an analysis is based on informed decision-making involving therapy-related considerations (evidence-based medicine, publications, guidelines, systematic reviews), as well as economic aspects involving individual therapy cost factors (direct, indirect, and intangible).

**Figure 5:**
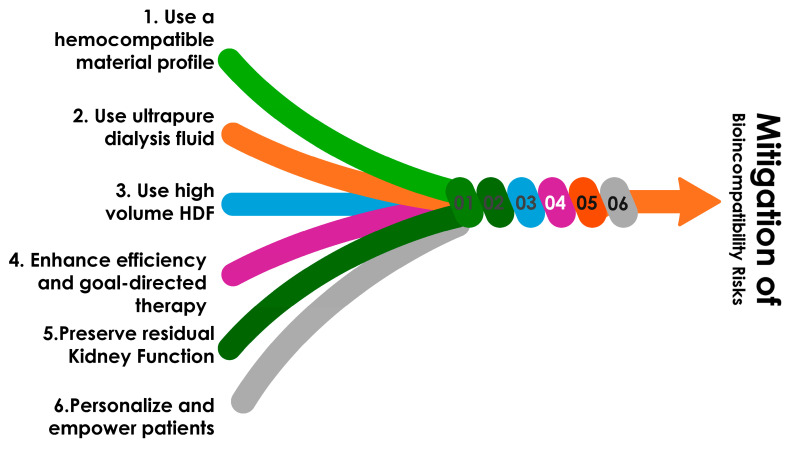
Proposed strategies to mitigate risk associated with bioincompatibility reactions associated with chronic hemodialysis.

To reduce the economic burden of hemoincompatibility associated with hemodialysis therapies, several strategies can be implemented. This is schematized in Figure 5.

### 5.1. Use of More Biocompatible Extracorporeal Components

There is a worldwide trend towards using synthetic high-flux hemodialyzers with more biocompatible membranes and higher performance. Additionally, research is ongoing to develop new synthetic polymer membranes with functionally modified membranes. For example, stabilizing PVP with vitamin E is currently used to reduce blood interactions and provide more hydrophilic properties [181,182,183,184,185]. Further modifications are being implemented or tested, such as substituting bisphenol plasticizers or modifying the surface charge by binding heparin sulfate or other components. Tubing sets are also benefiting from advancements. New polymers with more stable plasticizers (without phthalate) are being used, and attempts are being made to incorporate antithrombotic compounds directly into the polymer. Additionally, better engineering of tubing sets is being explored. This includes features like cassette systems to suppress the blood–air interface, but also tubing sets to provide more regular pathways without diameter restrictions or dead zones to prevent clotting activation [186]. Finally, research on roller blood pumps is ongoing to reduce the risk of plastic spallation and erythrocyte and cell damage.

### 5.2. Use of Ultrapure Dialysis Fluid

The use of ultrapure dialysis fluid to prevent toxic risk but also to prevent the passage of microbial-derived compounds such as LPS or muramyl dipeptides to blood, which are known as enhancers of hemoincompatibility reactions, is now universally recognized [187,188].

### 5.3. Utilize Convective-Based Therapies Such as High-Volume Hemodiafiltration

There is now sufficient evidence to show that high-volume hemodiafiltration is superior to high-flux HD. It offers higher efficiency in uremic solute removal and control, leading to superior outcomes with a 23% reduction in all-cause mortality [189,190]. Additionally, it demonstrates consistently higher biocompatibility with reduced inflammation markers.

### 5.4. Personalizing Treatment Prescriptions and Schedules

The personalization of kidney replacement therapy is a response to patient-centered care to fit with patient tolerance and patient needs. That consists in customizing session duration, frequency of sessions, and location of treatment (in-center, home, and self-care). Furthermore, tailoring electrolytic prescriptions to individual patient needs is also an important feature to implement [191,192].

### 5.5. Enhancing Patient Monitoring and Early Risk Detection

The use of remote monitoring devices and digital health technologies to detect potential risks earlier and improve patient outcomes is an interesting approach that warrants further investigation to assess its value [193].

### 5.6. Empowering Patients

Empowering patients to adopt healthier lifestyles, such as regular exercise, quitting smoking, and adopting better dietary habits, can improve outcomes. Increasing or facilitating treatment adherence by therapeutic educational workshops and more actively involving patients in their treatment plans have been shown to improve outcomes [194,195].

### 5.7. Using Additional Medications When Necessary

Administering medications to correct residual hemoincompatible reactions, such as anti-inflammatory drugs and complement blockers or inhibitors when needed, may be additional options.

### 5.8. Preserving Residual Kidney Function

Implementing strategies to reduce dialysis-induced systemic stress, both hemodynamic and non-hemodynamic, through more biocompatible modalities is crucial for preserving residual kidney function, as indicated earlier.

## 6. Conclusions

Significant progress has been made over the last few decades in reducing biological reactions associated with hemoincompatibility in hemodialysis-related therapies. These advancements include the extensive use of synthetic dialyzer membranes, new polymer materials in tubing sets, the ultra-purity of dialysis fluid and water, and more efficient therapies including high-flux HD and high volume HDF.

However, despite these advancements, residual hazards and serious consequences remain associated with the hemoincompatibility of dialysis systems. These include unpredictable acute life-threatening reactions and delayed dialysis-related diseases such as accelerated cardiovascular disease, accelerated aging, a compromised immune system, and the burden of kidney disease treatment.

These side effects need to be considered seriously in future research, as they have significant health and economic consequences for a therapy that is already very expensive.
